# Rare variants in *BMAL1* are associated with a neurodevelopmental syndrome

**DOI:** 10.1073/pnas.2427085122

**Published:** 2025-07-28

**Authors:** Vishnu Anand Cuddapah, Dechun Chen, Bumsik Cho, Rebecca Moore, Mohnish Suri, Hana Safraou, Frederic Tran-Mau-Them, Ashley Wilson, Jacqueline Odgis, Atteeq U. Rehman, Carol Saunders, Shiva Ganesan, Vaidehi Jobanputra, Stephen W. Scherer, Ingo Helbig, Amita Sehgal

**Affiliations:** ^a^Jan and Dan Duncan Neurological Research Institute, Texas Children’s Hospital, Houston, TX 77030; ^b^Division of Neurology and Developmental Neuroscience, Department of Pediatrics, Baylor College of Medicine, Houston, TX 77030; ^c^HHMI, University of Pennsylvania, Philadelphia, PA 19104; ^d^Chronobiology and Sleep Institute, Perelman School of Medicine, University of Pennsylvania, Philadelphia, PA 19104; ^e^Nottingham Clinical Genetics Service, Nottingham University Hospitals National Health Service Trust, Greater Nottingham NG5 1PB, United Kingdom; ^f^Laboratoire de Génomique médicale, Centre Hospitalier Universitaire Dijon-Bourgogne, Dijon 21000, France; ^g^Génétique des Anomalies du Développement Unité Mixte de Recherche 1231 INSERM Université de Bourgogne Franche-Comté, Dijon 21000, France; ^h^New York Genome Center, New York, NY 10013; ^i^Icahn School of Medicine at Mount Sinai, New York, NY 10029; ^j^Department of Pathology and Laboratory Medicine, Children’s Mercy–Kansas City and Departments of Pediatrics and Pathology, University of Missouri-Kansas City School of Medicine, Kansas City, MO 64108; ^k^Division of Neurology, The Epilepsy NeuroGenetics Initiative, Department of Biomedical and Health Informatics, Children’s Hospital of Philadelphia, Philadelphia, PA 19104; ^l^Columbia University Irving Medical Center, New York, NY 10032; ^m^The Centre for Applied Genomics, Program in Genetics and Genome Biology, The Hospital for Sick Children, Toronto, ON M5G 0A4, Canada; ^n^McLaughlin Centre and Department of Molecular Genetics, University of Toronto, Toronto, ON M5S 1A8, Canada; ^o^Department of Neurology, University of Pennsylvania, Perelman School of Medicine, Philadelphia, PA 19104

**Keywords:** BMAL1, neurodevelopmental disorder, circadian rhythms, developmental delay, *Drosophila*

## Abstract

Through international gene-matching efforts, we identified 10 individuals with ultrarare heterozygous variants, including 5 de novo variants, in *BMAL1*, a core component of the molecular clock. Instead of an isolated circadian phenotype seen with disease-causing variants in other molecular clock genes, all individuals carrying *BMAL1* variants surprisingly share a clinical syndrome manifest as developmental delay and autism spectrum disorder, with variably penetrant sleep disturbances, seizures, and marfanoid habitus. Variants were functionally tested in cultured cells using a *Per2*-promoter driven luciferase reporter and revealed both loss-of-function and gain-of-function changes in circadian rhythms. The tested *BMAL1* variants disrupted *PER2* mRNA cycling, but did not cause significant shifts in cellular localization or binding with CLOCK. Conserved variants were further tested in *Drosophila*, which confirmed variant-dependent effects on behavioral rhythms. Remarkably, flies expressing variant *cycle*, the ortholog of *BMAL1*, also demonstrated deficits in short- and long-term memory, reminiscent of the highly prevalent developmental delay observed in our cohort. We suggest that ultrarare variants in the *BMAL1* core clock gene contribute to a neurodevelopmental disorder.

## Significance

Children with neurodevelopmental disorders exhibit highly penetrant sleep and circadian dysfunction, but the underlying mechanisms are unclear. We asked whether a subset of individuals with neurodevelopmental disorders might have genetic variants in genes known to drive circadian rhythms. Through international collaboration, we identified ten individuals with very rare genetic variants in *BMAL1*, a core component of the molecular clock. These individuals exhibited overlapping signs and symptoms including developmental delay, autism spectrum disorder, and variably penetrant marfanoid features. We functionally tested the identified *BMAL1* variants in cell culture and in vivo and found disrupted BMAL1 function. These findings demonstrate that neurodevelopmental dysfunction can be driven by variation in circadian clock genes in a subset of individuals.

**Fig. 1.**
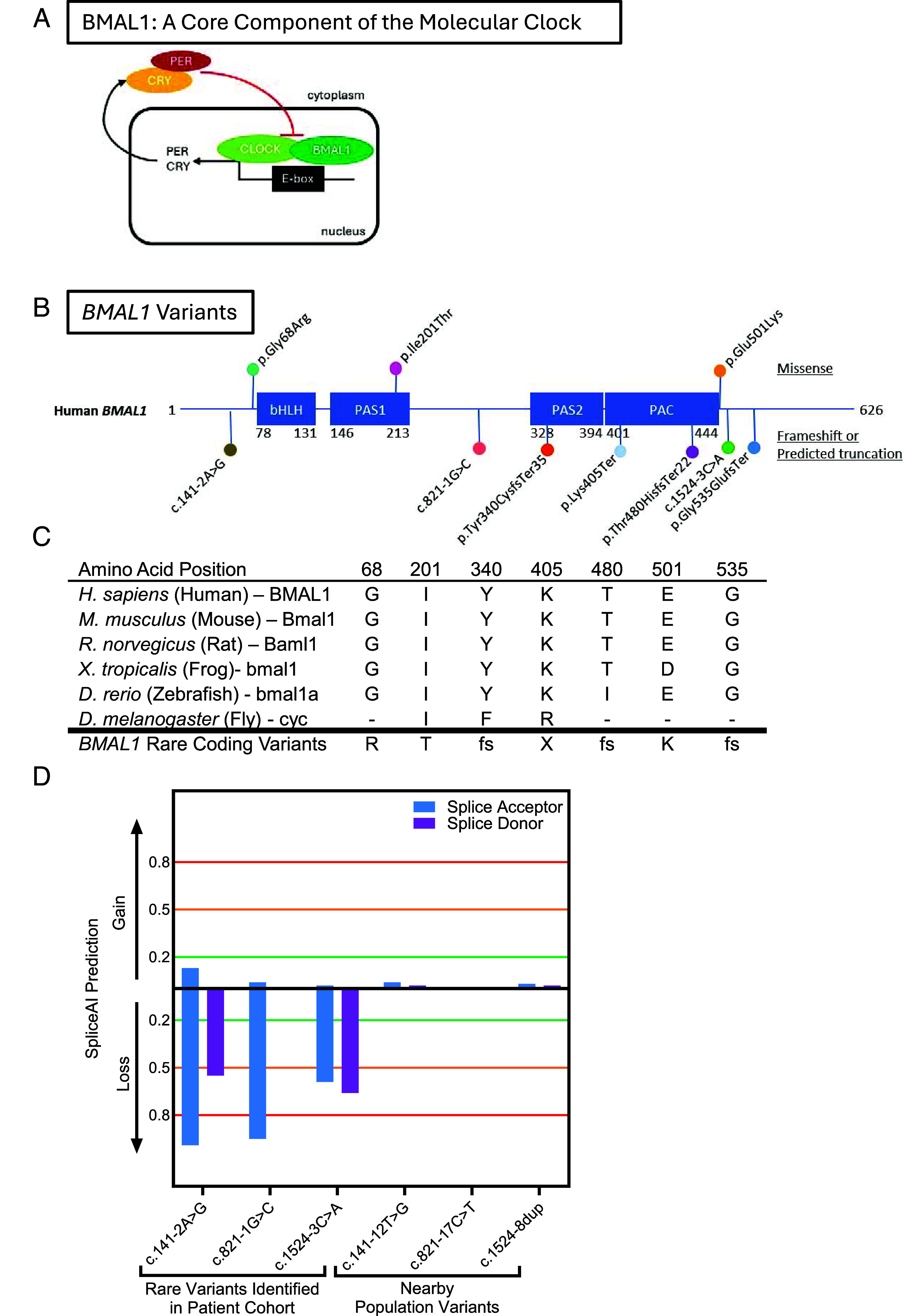
Characteristics of BMAL1 variants presented in this study. (*A*) The molecular clock is composed of a transcription–translation feedback loop. BMAL1 and CLOCK promote the transcription of the *PER* and *CRY* families of genes, and then are inhibited by those protein products over a period of 24 h. (*B*) Schematic of human BMAL1 protein with major protein domains depicted in *blue*. Missense variants are listed on top and frameshift or predicted truncations are depicted on bottom. (*C*) Amino acids implicated in this study demonstrate marked conservation across animal phyla. (*D*) SpliceAI was used to predict changes in splice acceptors or donors for the three rare splice site variants presented in this study, as well as three nearby common splice site variants. Rare variants are likely to lead to a loss of splice acceptor and/or donors. 0.8 indicates a stringent cutoff, 0.5 indicates a moderate cutoff, and 0.2 indicates a low cutoff value.

The molecular circadian clock is the primary mechanism allowing cells to maintain 24-h cycles. The mammalian molecular clock is composed of the transcription factors BMAL1 and CLOCK, which heterodimerize and bind to the E-box domain of the *PER* and *CRY* families of genes to promote their transcription (Fig. 1*A*). The PER and CRY proteins, in turn, heterodimerize and block the transcriptional activation induced by BMAL1 and CLOCK (Fig. 1*A*). This transcription–translation feedback loop takes approximately 24 h to complete a cycle and is the primary backbone of daily timekeeping.

Widespread adoption of clinical exome sequencing has led to the identification of variants in core components of the molecular clock resulting in Mendelian phenotypes. Variants in *PER2, PER3,* and *CRY1* have been implicated in advanced sleep phase disorder (1–3), while variants in *CRY1* cause delayed sleep phase disorder (4). As predicted, these variants in clock genes affect circadian rhythms including the timing of sleep, but do not have other associated phenotypic features.

Here, we identified rare variants in *BMAL1* in 10 individuals with a neurodevelopmental phenotype characterized by developmental delay and autism spectrum disorder. Through functional testing using a cell-based reporter and a small animal model, we find these variants perturb BMAL1 function and result in circadian and memory deficits.

## Results

### Ultrarare Variants in *BMAL1* Are Predicted to Perturb Function.

Through international collaboration facilitated by GeneMatcher (5) and review of published cohorts (6, 7), we identified 10 individuals with rare heterozygous variants in *BMAL1*, including five individuals with de novo variants. gnomAD, a large database of genetic sequencing data that captures natural variation throughout the population, reveals that *BMAL1* is a highly constrained gene that is intolerant to loss-of-function changes (pLI = 1) and missense variation (Z-score = 3.64), raising the possibility that rare nonsynonymous coding variants have functional consequences.

We identified 3 heterozygous missense ultrarare variants, as well as 7 heterozygous ultrarare variants predicted to introduce a frameshift and/or premature truncation (Fig. 1*B*). BMAL1 has four well-characterized protein domains including, a) a basic helix–loop–helix (bHLH) domain that enables binding to DNA, b) and c) PAS1 and PAS2 domains, which mediate heterodimerization with CLOCK (8), and d) a PAC domain that facilitates folding of the PAS domains. One of the missense variants, *BMAL1* NM_001297719.2:c.602 T>C; p.(Ile201Thr), occurs in the PAS1 domain (Fig. 1*B*) and is present twice in gnomAD, (v.4.1.0) with an allele frequency of 1.24e-6 and Combined Annotation Dependent Depletion (CADD) score of 23.5, suggesting it is likely deleterious (*SI Appendix*, Table S1). The other two missense variants, *BMAL1* NM_001297719.2:c.202G>A; p.(Gly68Arg) and *BMAL1* NM_001297719.2:c.1501G>A; p.(Glu501Lys), have no population frequency and CADD scores of 24.4 and 23.5, indicating they are also likely deleterious (*SI Appendix*, Table S1). The *BMAL1* p.(Glu501Lys) variant is particularly striking because it leads to the substitution of negatively charged glutamic acid with a positively charged lysine. Indeed, in silico analysis using AlphaMissense(9) indicates this variant is predicted to disrupt protein structure (score of 0.9724 with scores between 0.564 to 1 likely to be pathogenic).

One frameshift variant occurs in the PAS2 domain [NM_001297719.2:c.1019_1020del; p.(Tyr340CysfsTer35)], while the PAC domain contains a premature truncation [NM_001351824.2:c.1212dupT; p.(Lys405Ter)] and a frameshift variant [NM_001297719.2:c.1437dupC; p.(Thr480HisfsTer22)]; these variants are absent from the gnomAD v4.1.0 database and have no known population frequency. All coding variants occur at highly conserved residues (Fig. 1*C*). The NM_001297719.2:c.1604del; p.(Gly535GlufsTer) variant occurs closest to the C-terminus of the variants presented here and has no population frequency (*SI Appendix*, Table S1). All of the predicted truncating variants described here are located upstream of the last two exons of *BMAL1*, and therefore, are likely to be subjected to nonsense-mediated decay.

The splice-site variants are distributed throughout the gene including before the bHLH domain (NM_001351824.2:c.141-2A>G), before the PAS2 domain (NM_001297719.2:c.821-1G>C), and after the PAC domain (NM_001297719.2:c.1524-3C>A). We used SpliceAI, a deep-learning tool, to predict the functional consequences of these intronic variants (10). Using a stringent cutoff of 0.8, we found the c.141-2A>G and c.821-1G>C variants cause loss of splice acceptor sites (Fig. 1*D*). Additionally, using a moderate cutoff of 0.5, c.141-2A>G leads to a loss of a splice donor and c.1524-3C>A leads to loss of splice acceptor and donor sites (Fig. 1*D*). The c.141-2A>G variant was present only once in gnomAD for a frequency of 6.2e-7, and the other 2 splice-site variants had no population frequency. In contrast, nearby variants within the same intron with higher population frequencies had no predicted effect on splicing (Fig. 1*D*). For example, c.141-12 T>G, c.821-17C>T, and c.1524-8dup are present 7, 66, and 29 times in gnomAD, respectively, and are not expected to perturb splicing (Fig. 1*D*). Therefore, the ultrarare *BMAL1* intronic variants we present here are likely to affect splicing in contrast to more common variants.

### Rare Variants in *BMAL1* Are Associated with a Neurodevelopmental Phenotype.

For individuals harboring rare *BMAL1* variants, we found the most prevalent phenotypic characteristics were neurodevelopmental features. All the individuals for whom phenotypic information was available (8/8; 100%) exhibited developmental delay (*SI Appendix*, Tables S2 and S3). For at least four individuals (4/8; 50%) this manifested as global developmental delay, indicating that two or more domains of development were involved. Speech and motor skills were most often affected in the mild-to-moderate range. Seizures were noted in 50% of individuals (3/6) and autism spectrum disorder was noted in 6 individuals. Attention-deficit/hyperactivity disorder was noted in two individuals, as were heart murmurs. Given that *BMAL1* plays an important role in the molecular clock, we attempted to further characterize sleep or circadian phenotypes. We were able to obtain phenotypic information on sleep patterns in 7/10 individuals. Within this subcohort, 43% (3/7) exhibited sleep challenges.

Interestingly, 33% (2/6) of individuals for whom relevant history was available in this cohort exhibited a marfanoid habitus. In Individual six, this was described as a tall stature with dolichostenomelic features. An additional 33% (2/6) exhibited joint hypermobility with hypotonia. Thus, a musculoskeletal phenotype manifest as marfanoid habitus or joint hypermobility was present in 66% (4/6) of this cohort and may be a common feature of this clinical syndrome.

For the variants for which inheritance could be confirmed, 71% (5/7) were de novo (*SI Appendix*, Table S1). The *BMAL1* c.141-2A>G variant in Individual #1 was inherited from a mother who had an unremarkable medical history other than postpartum depression. Individual #1’s medical history was remarkable for a) an elder sister with learning difficulties and a history of infantile spasms, b) a maternal cousin with autistic symptoms and motor coordination difficulties, and c) another maternal cousin with learning difficulties and a mood disorder. These other family members were not available for genetic testing. The *BMAL1* p.(Lys405Ter) variant in Individual #6 was inherited from a father who had a history of learning disability and tics and was unable to read and write. Parental testing was not possible in Individuals #4 and #9 because the parents were deceased or not available and in Individual #10 because the child was adopted.

Other variants of unclear significance were identified in some of the individuals presented here but none provided a single unifying diagnosis (see details in *SI Appendix*, Table S4).

### Rare Heterozygous *BMAL1* Variants Perturb Protein Function.

To test the functional impact of the identified *BMAL1* variants, we used a cell-based reporter assay. A well-established target of BMAL1 is *Per2*, whose BMAL1-dependent rhythmic expression can be measured in cultured cells (11). Human U2OS cells stably expressing a *Per2*-promoter driven luciferase reporter (p*Per2*-dLuc) were previously created (12). Then, using CRISPR-Cas9 gene editing, we attempted to create heterozygous mutant lines that harbor each of the 10 *BMAL1* variants identified. Through multiple attempts, we were able to generate clonal lines for 9/10 variants. We were unable to introduce the *BMAL1* p.(Lys405Ter) variant due to lack of specific gRNA.

**Fig. 2.**
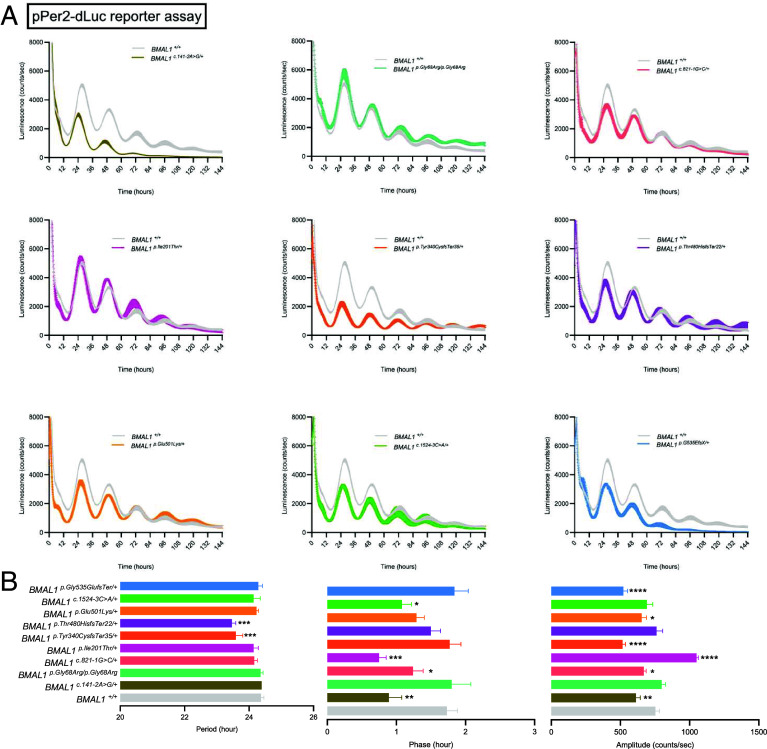
U2OS cells expressing *Per2*-dLuc reporter and *BMAL1* variants reveal altered *BMAL1* function. (*A*) Raw luminescence signal after dexamethasone synchronization was recorded for 6 d. The same genetic control condition *BMAL1^+/+^* is plotted in each trace to aid in comparison to variants. Traces indicate average values and thickness of the line depicts SE of the mean. n = 5 to 9 experiments/condition. (*B*) Circadian parameters calculated through BioDare2. One-way ANOVA with Benjamini, Krieger, and Yekutieli’s two-stage step-up method to control the false discovery rate for multiple comparisons made to the control genotype. *P < 0.05 **P < 0.01 ***P < 0.001. Data are presented as mean values ± SEM.

Circadian rhythms can be synchronized across individual U2OS cells by transient exposure to dexamethasone (11). We measured bioluminescence of *Per2*-dLuc for 6 d following dexamethasone treatment, and computed circadian period, phase, and amplitude using BioDare2 (13). As compared to mock transfected wild-type cells (*BMAL1^+/+^*), two heterozygous *BMAL1* frameshift variants, p.(Tyr340CysfsTer35) and p.(Thr480HisfsTer22), led to a significant shortening of the period from 24.4 h to 23.6 h and 23.5 h, respectively (Fig. 2). The 3 heterozygous splice-site *BMAL1* variants, c.141-2A>G, c.821-1G>C, and c.1524-3C>A, led to a phase shift. In addition, 6 variants led to a dampening of the magnitude of luminescence, including c.141-2A>G, c.821-1G>C, p.(Tyr340CysfsTer35), p.(Glu501Lys), and p.(Gly535GlufsTer); this is consistent with decreased *Per2* transcription and a loss-of-function mechanism. Interestingly the *BMAL1* p.(Ile201Thr) missense variant led to a significant enhancement of signal magnitude, consistent with a gain-of-function change. To more directly assess the strength of circadian cycling independent of changes in magnitude of expression, we normalized luminescence intensity to the nadir of the first 24 h for each variant and found the underlying amplitude of cycling was either preserved or enhanced as compared to control (*SI Appendix*, Fig. S1). Together, these data demonstrate that variant BMAL1 decreased *Per2* expression but did not decrease the amplitude of circadian cycling. We were unable to create a heterozygous *BMAL1* p.(Gly68Arg) line, but even in the homozygous state no phenotype was observed. In total, we observed that 8/9 of the rare *BMAL1* variants tested disrupted protein function.

**Fig. 3.**
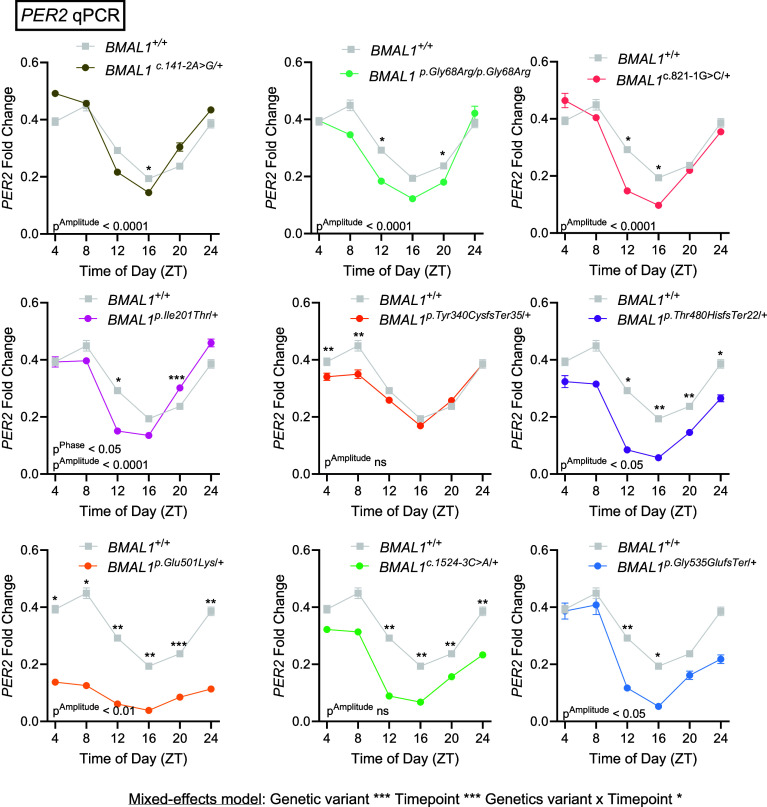
Altered *PER2* expression caused by BMAL1 variants. qPCR results *PER2* expression at six timepoints through the day in wild-type and variant BMAL1 U2OS cells. Daily oscillation of *PER2* mRNA in control cell is in gray and replotted in each trace to allow for comparison to variant lines. Circadian parameters were calculated through BioDare2 and significant results are listed in respective graphs. n = 3 samples/timepoint/condition. One-way ANOVA with Benjamini, Krieger, and Yekutieli’s two-stage step-up method to control the false discovery rate for multiple comparisons made to the control genotype. To compare mRNA levels independent of circadian metrics, a mixed-effects model with Geisser–Greenhouse correction and Dunnett’s multiple comparisons test were used. *P < 0.05 **P < 0.01 ***P < 0.001. Data are presented as mean values ± SEM.

The conversion of luciferin to oxy-luciferin by luciferase is an ATP-dependent process, so if mutations in *BMAL1* alter ATP levels, rhythms in luminescence might be affected. Therefore, we sought to directly measure *PER2* cycling. We synchronized U2OS cells containing *BMAL1* variants then quantified *PER2* mRNA cycling to assess for acute effects postsynchronization. Rhythm amplitude was calculated using BioDare2 (13) and revealed changes in rhythm amplitudes in 7/9 mutant lines tested (Fig. 3). A phase shift was again identified in U2OS cells harboring the *BMAL1* p.(Ile201Thr) missense variant (Fig. 3). Consistent with the decreased magnitude of luminescence from the *Per2*-dLuc assay, we identified decreased levels of *PER2* mRNA at various timepoints in cells containing *BMAL1* c.141-2A>G, c.821-1G>C, p.(Tyr340CysfsTer35), p.(Glu501Lys), and p.(Gly535GlufsTer) variants, as well as the p.(Gly68Arg)/p.(Gly68Arg), p.(Thr480HisfsTer22), and c.1524-3C>A conditions. Paralleling the increased magnitude of luminescence in the *Per2*-dLuc assay, the *BMAL1* p.(Ile201Thr) variant was the only to significantly increase *PER2* transcript levels at any timepoint. We sought to validate these results for another transcriptional target of BMAL1, *NR1D1*, and found similar results (*SI Appendix*, Fig. S3). The *BMAL1* p.(Ile201Thr) variant again increased the amplitude of *NR1D1* rhythms, while the p.(Tyr340CysfsTer35) variant decreased *NR1D1* mRNA. Note that the frequency and duration of sampling for RNA cycling experiments, relative to the luciferase assays above, makes it difficult to detect small changes in phase or period. These data are consistent with the *Per2*-dLuc reporter assay and provide evidence that all 9 rare *BMAL1* variants affect protein function such that the magnitude and/or cycling of *PER2* is disrupted within 24-h of synchronization.

### BMAL1 Expression and CLOCK Interaction in Mutant Cell Lines.

**Fig. 4.**
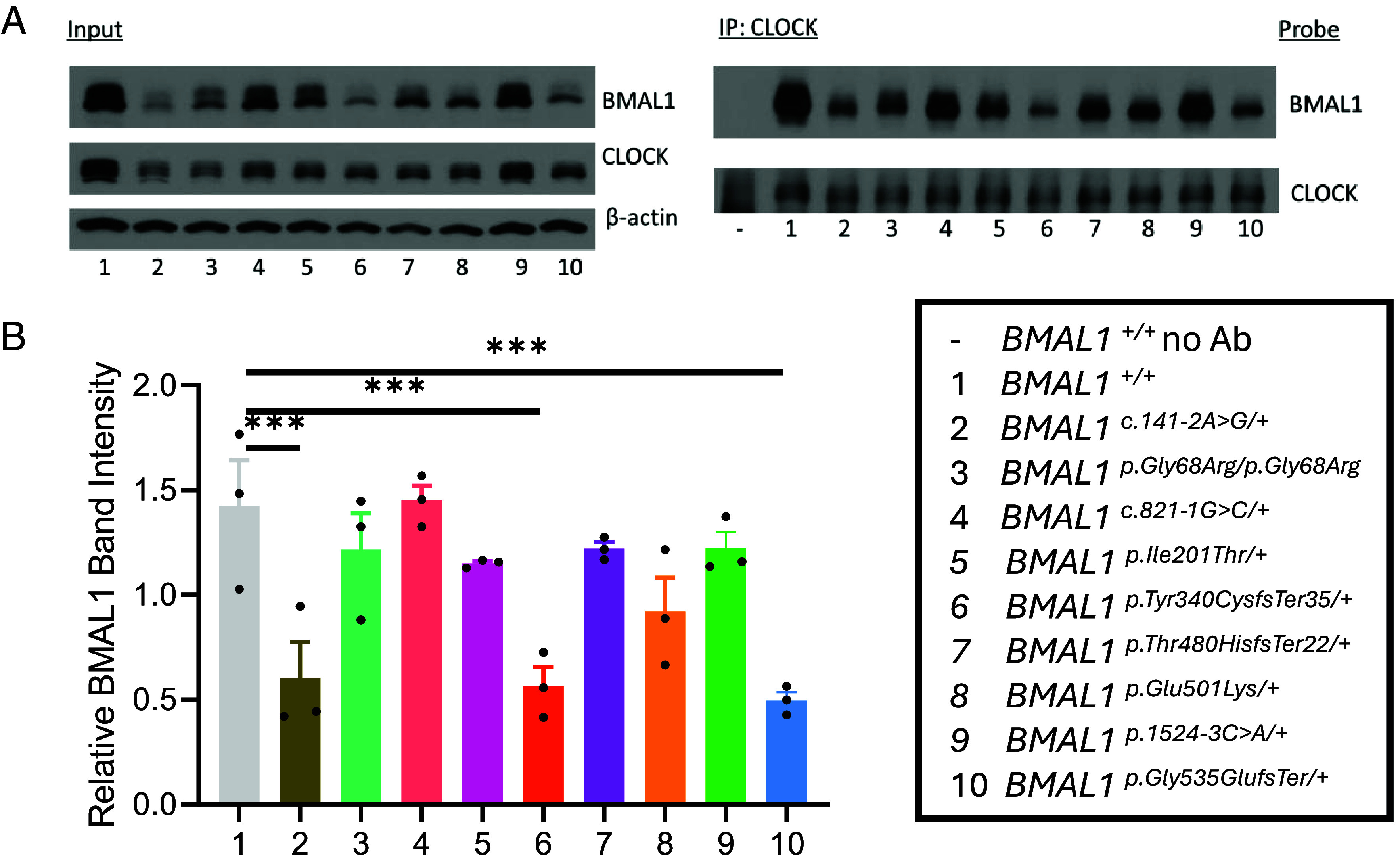
BMAL1 expression and interaction with CLOCK. (*A*) Representative immunoblots depicting expression of BMAL1 and CLOCK, as well as β-actin loading control, in wild-type, and variant BMAL1 cell lines (*Left)*. Immunoprecipitation of CLOCK and probing for BMAL1 demonstrates preserved protein–protein interactions (*Right*). (*B*) Quantification of BMAL1 band intensity in input as normalized to β-actin loading control reveals decreased BMAL1 expression caused by BMAL1 c.141-2A>G, p.(Tyr340CysfsTer35), and p.(Gly535GlufsTer) variants. n = 3 replicates/condition. One-way ANOVA with Holm–Sidak’s multiple comparisons test. ***P < 0.001. Data are presented as mean values ± SEM.

Effects of the rare *BMAL1* variants tested above on circadian cycling prompted us to ask whether these variants affected BMAL1 protein expression and interaction with CLOCK. The heterozygous BMAL1 c.141-2A>G, p.(Tyr340CysfsTer35), and p.(Gly535GlufsTer) variants led to a ~50% reduction of protein expression (Fig. 4), consistent with the variant mRNAs undergoing nonsense-mediated decay. While the *BMAL1* c.821-1G>C, p.(Thr480HisfsTer22), and p.1524-3C>A variants were also predicted to reduce BMAL1 expression, a normal amount of protein was observed. As expected, the missense variants did not change BMAL1 total protein expression. Coimmunoprecipitation studies of CLOCK and BMAL1 demonstrated that there were no changes in binding (Fig. 4). Additionally, immunohistochemistry of U2OS cells harboring these rare BMAL1 variants did not reveal mislocalization of BMAL1 or CLOCK (*SI Appendix*, Fig. S2). Overall, the c.141-2A>G, p.(Tyr340CysfsTer35), and p.(Gly535GlufsTer) variants decrease BMAL1 expression, and none of the variants markedly impair binding to CLOCK or cellular localization.

### Flies Expressing Orthologous *BMAL1* Variants Exhibit Altered Behavioral Rhythms.

**Fig. 5.**
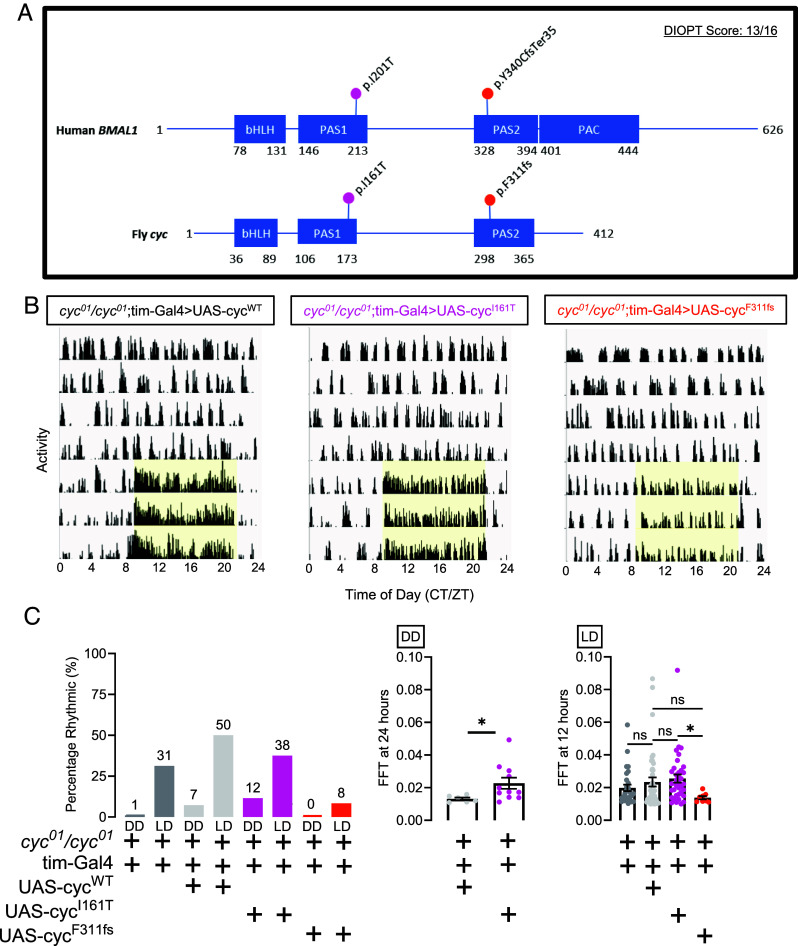
Flies harboring orthologous *cyc* variants exhibit circadian alterations. (*A*) *cycle* is the fly ortholog of *BMAL1* with a DRSC Integrative Ortholog Prediction Tool (DIOPT) score of 13, indicating high homology. The BMAL1 p.I201T variant in the PAS1 domain corresponds with the cycle p.I161T variant in the PAS1 domain. The BMAL1 p.Y340CfsTer35 variant in the PAS2 domain corresponds with the cycle p.F311fs variant in the PAS2 domain. (*B*) Representative actograms demonstrating that loss-of-function *cyc^01^/cyc^01^* flies lack rhythmic behavior in total darkness, but when wild-type *cyc* (UAS-cyc^WT^) or *cyc* p.I161T (UAS-cyc^I161T^) is expressed, behavioral rhythmicity is improved in light–dark cycles. *Yellow shading* indicates presence of light and resumption of ZT9-9 light:dark cycle. (*C*) Quantification of behavioral rhythmicity. *Left*: percentage of flies demonstrating rhythmic behavior as measured by an FFT value above 0.01. *Middle* and *Right:* FFT values for flies with rhythmic behavior in total darkness (DD) and light–dark cycles (LD). Note that rhythmic flies were rarely seen in the *cyc^01^/cyc^01^*;tim-Gal4 and *cyc^01^/cyc^01^*;tim-Gal4> UAS-cyc^F311fs^ conditions in DD, so FFT values are not provided for the *Middle* panel. n = 82-96 flies/condition. The Mann–Whitney test was used when comparing two groups given lack of normal distribution. The Kruskal–Wallis test was used when comparing three or more groups with Dunn’s multiple comparisons test given lack of normal distribution. *P < 0.05. Data are presented as mean values ± SEM.

Given that the rare *BMAL1* variants presented here perturb protein function, we sought to understand how these variants might affect behavior by modeling variants in the *Drosophila* ortholog *cycle* (*cyc*). *BMAL1* and *cyc* are highly homologous with a DIOPT score of 13 (Fig. 5*A*). As intronic splice-site variants, PAC domain variants, and C-terminal variants in *BMAL1* are not well conserved in *cyc*, we focused our attention on the *BMAL1* p.(Ile201Thr) variant, which increased circadian amplitude consistent with a gain-of-function mechanism, and the *BMAL1* p.(Tyr340CysfsTer35) variant, which decreased circadian amplitude consistent with a loss-of-function mechanism. Protein alignments demonstrated that *BMAL1* p.Ile201 is orthologous to *cyc* p.Ile161, and *BMAL1* p.Tyr340 is orthologous to *cyc* p.Phe311. Tyrosine (Y) and phenylalanine (F) are closely related amino acids with hydrophobic side chains differing by a single hydroxyl group. Thus, we generated *cyc* variants p.I161T and p.F311fs, which are equivalent to the corresponding *BMAL1* variants, in a *cyc* transgenic construct. We then created flies harboring wild-type *cyc* under UAS control (UAS-cyc^WT^), and mutant lines harboring either *cyc* p.I161T or *cyc* p.F311fs under UAS control (UAS-cyc^I161T^ and UAS-cyc^F311fs^ respectively). These flies were then crossed to homozygous *cyc* mutant flies (*cyc^01^/cyc^01^*) (14), which are arrhythmic.

In complete darkness (DD), *cyc^01^/cyc^01^* mutants expressing the *tim*-Gal4 driver were arrhythmic using a fast Fourier transform (FFT) value of 0.01 as a cutoff for rhythmicity (Fig. 5*C*). The *tim*-Gal4 driver targets cells expressing molecular clock genes, including the ~240 brain clock neurons. When wild-type *cyc* or *cyc* p.I161T expression is driven with the *tim*-Gal4 driver, although full rescue is not achieved, some flies become rhythmic in constant darkness (Fig. 5*C*). FFT values were marginally, though significantly, higher in rhythmic flies expressing *cyc* p.I161T in DD, relative to those expressing wild type *cyc*, consistent with a gain-of-function mechanism and cell culture results (Fig. 5*C*). In contrast, driving *cyc* p.F311fs expression in DD did not improve rhythmicity, consistent with a loss-of-function mechanism and cell culture results (Fig. 5*C*). Upon shifting from DD to a 12-h light:12-h dark cycle (LD), only 31% of *cyc^01^/cyc^01^* mutants exhibit rhythmic behavior (Fig. 5*C*), consistent with previous results demonstrating impaired behavioral rhythmicity in *cyc* mutants even in cycling light:dark conditions (14). A greater percentage of mutant flies expressing wild-type *cyc* or *cyc* p.I161T demonstrated rhythmic activity in light:dark conditions (Fig. 5 *B* and *C*). Only 8% of mutant flies expressing *cyc* p.F311fs were rhythmic in light:dark cycles (Fig. 5 *B* and *C*), which was less than levels seen in *cyc^01^/cyc^01^* mutant flies, and therefore consistent with a severe loss-of-function or possibly dominant negative mechanism. Overall, these in vivo experiments largely recapitulate both gain- and loss-of-function mechanisms observed in culture.

### Orthologous *BMAL1* Variants Impair Memory.

**Fig. 6.**
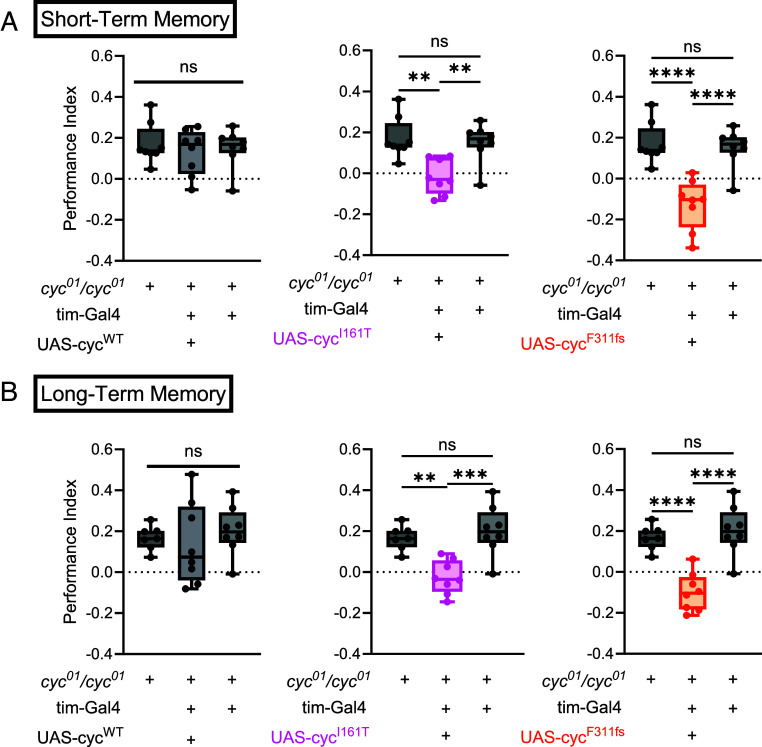
Short- and long-term memory is impaired in flies expressing variant *cycle.* (*A*) Quantification of short-term memory in *cyc^01^/cyc^01^* flies expressing wild-type *cyc* (UAS-cyc^WT^), *cyc* p.I161T (UAS-cyc^I161T^), or *cyc* p.F311fs (UAS-cyc^F311fs^) using the tim-Gal4 driver. *cyc* p.I161T and *cyc* p.F311fs disrupts short-term memory. (*B*) Quantification of long-term memory in *cyc^01^/cyc^01^* flies expressing wild-type *cyc*, *cyc* p.I161T, or *cyc* p.F311fs using the tim-Gal4 driver. *cyc* p.I161T and *cyc* p.F311fs impairs long-term memory. n = 7 to 8 replicates/condition. One-way ANOVA with Tukey’s multiple comparisons test. Data are presented as box and whiskers and showing all points.

In our cohort, we found that, for all the individuals for whom clinical information was available, all experienced developmental delay. Therefore, we sought to understand whether these variants disrupt short- and long-term appetitive memory. We found that *cyc^01^/cyc^01^* mutant flies were able to form normal short- and long-term memory (Fig. 6 *A* and *B*). Driving wild-type *cyc* with the *tim*-Gal4 driver in *cyc^01^/cyc^01^* mutant flies did not significantly change memory, but driving either *cyc* p.I161T or *cyc* p.F311fs significantly suppressed short- and long-term memory as compared to controls. Thus, *cyc* gain-of-function through *cyc* p.I161T expression improves circadian rhythms, but impairs memory. On the other hand, *cyc* p.F311fs is deficient in its ability to drive circadian rhythms and also results in impaired memory, supporting the idea that this variant exerts a toxic dominant-negative effect.

## Discussion

We identified 10 individuals with neurodevelopmental features, sleep dysfunction, and musculoskeletal symptoms associated with ultrarare variants in *BMAL1*. Modeling 9 of these variants in cell culture demonstrates disrupted BMAL1 transcriptional activity. Additional study of 2 of these variants conserved in *Drosophila* revealed both gain-of-function and dominant negative changes that affect behavioral rhythms and learning and memory. Thus, pathogenic variants in core clock genes can contribute to neurodevelopmental phenotypes.

We find that all 9 of the rare *BMAL1* variants tested affected protein function to varying degrees. The 3 *BMAL1* splice site variants spread throughout the gene advanced the circadian phase and decreased *PER2* transcription as measured through the *PER2*-dLuc reporter assay, consistent with a loss-of-function mechanism (Fig. 2). The decrement in *PER2* mRNA expression was confirmed within the first 24 h after synchronization predominantly at or after ZT12 (Fig. 3). The 3 missense variants produced the most varied effects: *BMAL1* p.(Gly68Arg)/p.(Gly68Arg) and p.(Glu501Lys) produced partial loss-of-function changes, while the p.(Ile201Thr) variant increased both molecular and behavioral rhythmicity, consistent with a gain-of-function mechanism. Given that the *BMAL1* p.(Ile201Thr) residue is in the PAS1 domain, which directly contacts CLOCK, it is possible that the p.(Ile201Thr) variant enhances dimerization with CLOCK to increase *PER2* transcription. Alternatively, given that we did not detect enhanced dimerization, it may decrease interaction with PER proteins. While *BMAL1* p.(Ile201Thr) enhanced circadian rhythmicity it, however, reduced memory consolidation (Fig. 6). The *BMAL1* p.(Gly68Arg)/p.(Gly68Arg) variant produced the weakest phenotype even in the homozygous state. Finally, the 3 frameshift variants all decreased *PER2* transcription consistent with loss-of-function. The *BMAL1* p.(Tyr340CysfsTer35) and p.(Thr480HisfsTer22) variants, occurring in the PAS2 and PAC domains, shortened the circadian period and therefore may impair interaction with CLOCK, although such impairment is likely small as it was not detected in our co-IP experiment. Both the advancement of circadian phase associated with splice site variants and the shortening of circadian period due to frameshift shift variants can be caused by decreased PER2 protein levels, as has been previously suggested (15). As in the case of p.(Ile201Thr), the p.(Tyr340CysfsTer35) and p.(Thr480HisfsTer22) variants could also affect interaction with PER/CRY, in this case increasing interaction to promote feedback and thereby decrease *PER2* RNA levels. In summary, our cell culture results reveal that rare *BMAL1* variants cause both loss-of-function and gain-of-function changes.

When attempting to rescue the *cyc^01^* mutant phenotype by driving wild-type *cyc* (UAS-cyc^WT^) using the tim-Gal4 driver, we did not observe a full rescue (Fig. 5). This is similar to previous attempts to rescue core clock components including *per*, *tim*, and *Clk* using neuronal and clock drivers that have failed to provide a full rescue of locomotor rhythms (16, 17). A possible explanation is either too high or too low Gal4 expression, which then would affect molecular rhythms. Beyond molecular rhythms, *cyc* plays an important role in non-clock-mediated cellular morphogenesis. If *cyc* is not rescued to wild-type levels in clock neurons early in development, then axonal projections are abnormal and predicted to limit communication with downstream partners (18). We note too that the developmental timing and spatial expression of *tim*-Gal4 could be somewhat different from that of endogenous *cyc.* The lack of a full rescue we observe is less likely due to rhythmic *tim*-Gal4 expression, as the Gal4 protein is expected to be stable and not cycle.

Nevertheless, our behavioral data from flies support the pathogenicity of conserved *BMAL1* variants. Consistent with our cell culture results, the *cyc* p.I161T variant improved rhythmicity over control levels suggesting a gain-of-function mechanism. Modeling of the p.(Tyr340CysfsTer35) variant in the fly (*cyc* p.F311fs) led to a reduction in the percentage of rhythmic flies in light–dark conditions and impaired memory as compared to loss-of-function mutants, possibly consistent with a toxic dominant-negative effect. Here, the loss-of-function change in cell culture is caused by haploinsufficiency after nonsense mediated decay, whereas the toxic dominant-negative effect in vivo is likely due to forced expression of a protein fragment. Both gain-of-function and dominant-negative variants impaired short- and long-term memory in the fly. In addition to their role in the molecular clock, both CYC and BMAL1 play a role in non-clock-mediated cellular and molecular mechanisms. For example, loss of *cyc* disrupts neuronal projections in clock neurons (18), and BMAL1 modulates the activity of other transcription factors, including ETS and N-MYC (19). Thus, it remains unknown if the memory impairment we observe results from a molecular timekeeping effect of *cyc* or nonrhythmic transcriptional regulation of genes involved in development and/or memory.

While common population variants in *BMAL1* have been associated with a variety of human disorders, we now implicate rare heterozygous pathogenic variants in *BMAL1* as a cause of a Mendelian neurodevelopmental disorder. Genome-wide association studies (GWAS) reveal that variants in *BMAL1* are associated with insulin resistance (20, 21), cardiovascular health (21, 22), prostate cancer (23), lung cancer (24), chronotype (25), sociability (26), and neurodegeneration (27). These common variants may affect BMAL1 function through unclear mechanisms, and likely lead to relatively small effects on associated phenotypes. In contrast, the rare variants presented here have no, or little, population frequency (*SI Appendix*, Table S1), and were predicted to perturb BMAL1 function through in silico analyses (*SI Appendix*, Table S1 and Fig. 1*C*). Indeed, functional testing in cultured cells, and flies, confirms these analyses (Fig. 2).

Examination of DECIPHER (Database of Chromosomal Imbalance and Phenotype in Humans Using Ensembl Resources) (28) for copy number variants (CNVs) reveals 11 heterozygous microdeletions ranging in size from 2.80 Mb to 134.18 Mb. These CNVs are de novo and associated with small size at birth (6/11), intellectual disability (4/11), and arachnodactyly (2/11), among other dysmorphic features. This cohort includes an individual with a heterozygous, de novo 4.26 Mb microdeletion involving *BMAL1* and associated with autism spectrum disorder and arachnodactyly, a marfanoid feature. While these features might be consistent with the cohort we present here, microdeletions involving *BMAL1* also include adjacent genes implicated in neurodevelopmental syndromes and sensitive to haploinsufficiency including *RRAS2* and *SOX6*.

Consistent with our culture findings, heterozygous *cyc* and *Bmal1* mutant animals have been previously demonstrated to exhibit a spectrum of phenotypes. Heterozygous *cyc^0^*^/+^ mutant flies exhibit behavioral rhythms, but the period of these rhythms is about 1-h longer than control flies (14). Heterozygous *Bmal1*^+/-^ mice, similarly, exhibit intact behavior rhythms (29) with subtle phenotypes. Autism-like behaviors, including altered vocalizations, deficits in socialization, and anxious behavior have now been identified in *Bmal1*^+/-^ mice (30). While *Bmal1*^+/-^ mice exhibit intact behavioral rhythms, mice heterozygous for a C-terminus truncated Bmal1 (*Bmal1*^+/GTΔC^) exhibit a gradual dampening of rhythms through a presumed dominant-negative mechanism (31). The BMAL1 p.(Gly535GlufsTer)/+ variant we present here mirrors this C-terminus truncated allele most closely and also demonstrates a clear dampening of rhythms (Fig. 2). However, the phenotype could just reflect loss-of-function, given the ~50% decrease in BMAL1 expression likely due to nonsense mediated decay (Fig. 4), leading to haploinsufficiency.

We find that 3/6 individuals, for whom history was available, exhibited seizures (*SI Appendix*, Tables S2 and S3). Electrically induced seizures are more likely to occur with less current injection in Bmal1 knockout mice, indicating increased seizure susceptibility. The drivers of increased seizure risk, and whether this occurs through molecular clock-dependent or -independent mechanisms, remain unclear. BMAL1 has tissue-specific functions independent of molecular clock function (32), and this may perhaps provide an explanation for the musculoskeletal phenotypes, including marfanoid habitus, seen in our cohort.

Sleep difficulties are highly prevalent in children with autism spectrum disorder, affecting up to 80% (33, 34). The nature of these sleep challenges is variable, and may include difficulty with sleep onset, maintenance, consolidation, timing, and/or quantity. Monogenic etiologies for alterations in sleep timing (1–4) and quantity (35–39) are rapidly being identified, largely through genetic sequencing of families exhibiting strong sleep or circadian phenotypes. Several variants affecting sleep timing occur in well-characterized circadian clock genes, including *PER2* (1), *PER3* (2), *CRY1* (4), and *CRY2* (3). Perhaps unsurprisingly, variants in *PER2* have now been identified in people with autism spectrum disorder with variable sleep phenotypes, including sleep timing (40). This begs a question related to causation: Is it possible that sleep dysfunction early in development can cause neurodevelopmental signs and symptoms related to autism spectrum disorder? Correlations have been drawn between the severity of autistic behaviors and sleep/circadian dysfunction (34). In our own cohort, given that *BMAL1* is a core component of the molecular clock, we hypothesized that there would be a high likelihood of sleep or circadian dysfunction. Instead, we found that sleep-related diagnoses were rarely made in children with autism spectrum disorder, and only 3 of 7 individuals in whom the information was available reported sleep difficulty. As has been previously reported, although few children with autism spectrum disorder received sleep-related diagnoses, sleep-related disturbances may be more common (40) and possibly consistent with an underdiagnosis.

An exciting corollary of our findings is the possibility that early and aggressive treatment of sleep or circadian disruption might decrease the severity of neurodevelopmental disease. We find that disruption of a core component of the molecular clock causes memory impairment. Future studies focused on understanding whether improvement of behavioral rhythmicity can improve neurodevelopment are warranted. If correction of circadian behaviors like sleep can improve neurodevelopment, this has broad applicability to neurodevelopmental disorders beyond those caused by pathogenic variants in molecular clock genes.

## Materials and Methods

### Cell Culture.

U2OS cells were cultured in Dulbecco’s modified Eagle’s medium supplemented with 10% FBS, 1% L-glutamine, and 1% penicillin-streptomycin (Thermo Fisher Scientific) at 37 °C under 5% CO2. *Mycoplasma* testing was performed and found to be negative.

### Generation of U2OS Cells Expressing Per2 Promoter-Driven Luciferase and BMAL1 Variants.

As previously reported (11, 12), the p*Per2*-dLuc lentiviral construct was gifted by Andrew C. Liu at the Department of Physiology and Functional Genomics, University of Florida. U2OS cell reporter lines stably expressing p*Per2*-dLuc were generated using lentivirus-mediated gene delivery and established transduction protocols (41).

U2OS cells stably expressing p*Per2*-dLuc were then used for knock-in edits. Single guide RNA (sgRNA) and single-stranded oligodeoxynucleotides (ssODNs) were designed and transfected by Synthego to generate edited lines also expressing p*Per2*-dLuc. The Inference of CRISPR Edits tool (Synthego) was used to analyze the efficiency of CRISP-mediated edits. All sgRNA, ssODNs, and PCR primers to confirm edits are provided in *SI Appendix*, Table S5.

### Bioluminescence Recording/LumiCycle Assay.

Real-time bioluminescence of Per2 or Bmal1 dLuc reporter U2OS cells after synchronization with 1 µM Dexamethasone (Sigma) were monitored using a LumiCycle luminometer (Actimetrics, Wilmette, IL, United States) as previously described (42).

### qPCR.

Total RNA was extracted from all cell lines at 6 different time points using TRIzol reagent (Thermo Fisher Scientific). RNA was reverse transcribed to generate cDNA using a High-Capacity cDNA Reverse Transcription kit (Thermo Fisher Scientific). qPCR was performed on a ViiA 7 Real-Time PCR System (Applied Biosystems) using SYBR Green PCR master mix (Thermo Fisher Scientific). GAPDH was used as a control. Primers (5′ to 3′) for qPCR used in the study are as follows:

Per2_Forward: GGATGCCCGCCAGAGTCCAGAT

Per2_Reverse: TGTCCACTTTCGAAGACTGGTCGC

NR1D1_Forward: CTGCCAGCAATGTCGCTTCAAG

NR1D1_Reverse: TGGCTGCTCAACTGGTTGTTGG

GAPDH_Forward: GTCTCCTCTGACTTCAACAGCG

GAPDH_Reverse: ACCACCCTGTTGCTGTAGCCAA

### BMAL1 and CLOCK Immunostaining.

Cells were cultured on Nunc™ Lab-Tek™ II CC2™ Chamber Slide System (Thermo Scientific Catalog # 12-565-2), and then fixed with 4% paraformaldehyde (PFA) 30 min at RT, permeabilized by washing 0.3% Triton X-100 in PBS 3 times (5 min a time). Cells were blocked using 3% NDS in PBS (blocking buffer) for 20 min RT. Cells were incubated with primary Abs in blocking buffer at RT for 1 h. Mouse anti-BMAL1 (Santa Cruz) and rabbit anti-CLK (Abcam) were used. Cells were washed with PBST (PBS + 0.1% Tween-20) for three times (10 min a time). Cells were then incubated with the following secondary Abs for 1 h: goat anti-Mouse IgG (H + L) Highly Cross-Adsorbed Secondary Antibody, Alexa Fluor™ Plus 488 (Thermo Fisher Scientific) and Goat anti-Rabbit IgG (H + L) Highly Cross-Adsorbed Secondary Antibody, Alexa Fluor™ Plus 594 (Thermo Fisher Scientific). Cells were then washed with PBST 3 × 10 min, and finally once with 1X PBS. Cells were then mounted using mounting medium (VECTASHIELD Antifade Mounting Medium with DAPI, Vector Laboratories). The slide was cured overnight at room temperature in the dark and imaged the following day under a confocal microscope (Leica Microsystems).

### Coimmunoprecipitation and Immunoblotting.

Cells were lysed with ice-cold lysis buffer containing 10 mM Tris-Cl pH7.5, 100 mM NaCl, 5 mM EDTA, 1% Triton X-100 and 0.05% SDS, and Protease inhibitor cocktail (Roche Diagnostics). Total protein concentration was measured using a DC Protein Assay kit (Bio-Rad). 1 µg of rabbit anti-CLK IgG (Abcam) and 50 µL of washed Protein A DynaBeads (Thermo Fisher Scientific) were incubated with 500 µg of total protein for 4 h at 4 °C, and then washed 6 times with ice-cold washing buffer (10 mM Tris-Cl pH7.5, 150 mM NaCl, 5 mM EDTA, 0.4% Triton X-100 and 0.05% SDS, Protease inhibitor cocktail). Co-IP samples and 10 µg input samples were loaded into wells of 15-well 4 to 12% Tris-Glycine precast gels (Thermo Fisher Scientific). Protein was separated through SDS-PAGE, transferred onto a nitrocellulose membrane, and incubated with primary antibodies including mouse anti-BMAL1 monoclonal antibody (Santa Cruz), rabbit anti-CLK antibody (Abcam) and mouse anti-βActin antibody (R&D Systems), and horseradish peroxidase conjugated secondary antibodies (Jackson ImmunoResearch). Following chemiluminescence, blots were exposed to X-ray film. Images were digitized using a scanner then imported into Image J for processing and quantification of blot intensity. In Image J, images were converted to grayscale and regions of interest were drawn around each band to measure intensity. Relative BMAL1 band intensity was calculated by dividing the BMAL1 band intensity by the β-actin band intensity for each individual sample.

### Mutagenesis of cyc and UAS-cyc Fly Generation.

For generation of UAS-cyc^WT^, UAS-cyc^I161T^, and UAS-cyc^F311fs^ flies, a vector with a *cyc* cDNA sequence was obtained from DGRC (GM02625) and used as a template. To create the *cyc* p.I161T and *cyc* p.F311fs mutations, we used a mutagenesis kit (NEB; E0554S) with the primers listed below. Mutated *cyc* sequences were validated with Sanger sequencing in the University of Pennsylvania DNA core facility. Wild type or mutated *cyc* cDNA were then subcloned to pUASt-AttB vector (DGRC 1419), and transgenic flies were generated by BestGene Inc. (Chino Hills, CA) with the PhiC31 injection method.

cyc_Forward: GGGCTCGAGATGGAAGTTCAGGAGTTCTGCG

cyc_Reverse: GGGTCTAGATTATAAGAACACGGAATTCTTGGCG

I161T_Forward: TCCGAAGGACACCGGCAAGGTTAAG

I161T_Reverse: TGCAGGACGTCGAAC

F311fs_Forward: TCTATCTCGCGCCACTCCGG

F311fs_Reverse: GCACGTGCCGTATGTTCGGGTGATTG

### *Drosophila* Memory Assays.

Appetitive conditioning was performed as previously described (43, 44).

For short-term memory assays, 4- to 7-d-old mixed-sex population of ~100 flies were starved (on 2 mL of 2% agar in vials) for 18 h and trained in a dark room with red light at 25 °C and 70% relative humidity. All experiments were conducted under vacuum pressure in a memory wheel, which can conduct 4 individual experiments at once. In brief Whatman paper, cut into 1-inch squares were soaked in either 1.5 M sucrose or milliQ water. Flies were exposed to odor A + sucrose for 2 min, followed by clean air for 30 s, and odor B + water for 2 min followed by clean air for 30 s. Following training, flies were immediately tested for short term memory. During short term memory training, flies were exposed to both odors at once for 2 min. Flies were then collected, placed onto complete media, and counted. Flies remained on complete media for 3 to 5 h following training and testing.

For long-term memory assays, the same flies trained and tested in short term memory were restarved on 2% agar (as above) for 18 h and tested for long term memory. Flies were exposed to both odor A and B for 2 min and collected depending on the choice that was made. Flies were then counted and discarded.

In both short-term and long-term memory assays, odor A = 3 mm odor cups supplemented with 80 uL of 3-octanol (1:80) and odor B = 4-methylcyclohexanol (1:200) in paraffin oil. Choice index (CI) was calculated as the number of flies selecting CS+ odor minus the number of flies selecting CS- odor divided by the total number of flies. Each CI is averaged with its reciprocal training counterpart to determine the Performance Index (PI), which minimizes nonassociative effects.

### Quantification and Statistical Analyses.

For datasets involving 2 groups and not normally distributed, a Mann–Whitney test was implemented to detect statistical differences. For datasets involving three or more groups and not normally distributed, we used a Kruskal–Wallis test with Dunn’s correction for multiple comparisons. For datasets involving three or more groups and reasonably assumed to be approximately normally distributed, we used a one-way ANOVA with correction for multiple comparisons.

To identify quantify circadian period, phase, and amplitude in the *Per2*-dLuc assays, we used BioDare2 to implement a Fast Fourier Transform (FFT-NLLS)(13)

For the qPCR dataset, BioDare2 was again implemented to calculate circadian parameters. For identification of differences between groups (Genetics versus Timepoint), a mixed-effects model with a Geisser–Greenhouse correction was used, as sphericity was not assumed, as well as Dunnett’s multiple comparisons test.

To visually represent that data, mean and SE of the mean were used. Additional details regarding statistical testing, sample size, and *P*-values are provided in the Figure Legends. GraphPad Prism was used for all statistical analyses.

## Supplementary Material

Appendix 01 (PDF)

## Data Availability

All study data are included in the article and/or *SI Appendix*.

## References

[1] K. L. Toh , An hPer2 phosphorylation site mutation in familial advanced sleep phase syndrome. Science **291**, 1040–1043 (2001). 11232563 10.1126/science.1057499

[2] L. Zhang , A PERIOD3 variant causes a circadian phenotype and is associated with a seasonal mood trait. Proc. Natl. Acad. Sci. U. S. A. **113**, 1536 (2016). 10.1073/pnas.1600039113 PMC4801303 26903630

[3] A. Hirano , A cryptochrome 2 mutation yields advanced sleep phase in humans. eLife **5**, e16695 (2016). 10.7554/eLife.16695. 27529127 PMC5398888

[4] A. Patke , Mutation of the human circadian clock gene CRY1 in familial delayed sleep phase disorder. Cell **169**, 203–215.e13 (2017). 28388406 10.1016/j.cell.2017.03.027 PMC5479574

[5] N. Sobreira, F. Schiettecatte, D. Valle, A. Hamosh, GeneMatcher: A matching tool for connecting investigators with an interest in the same gene. Hum. Mutat. **36**, 928–930 (2015). 26220891 10.1002/humu.22844 PMC4833888

[6] R. K. C. Yuen , Whole genome sequencing resource identifies 18 new candidate genes for autism spectrum disorder. Nat. Neurosci. **20**, 602–611 (2017). 28263302 10.1038/nn.4524 PMC5501701

[7] J. Kaplanis , Evidence for 28 genetic disorders discovered by combining healthcare and research data. Nature **586**, 757–762 (2020). 33057194 10.1038/s41586-020-2832-5 PMC7116826

[8] N. Huang , Crystal structure of the heterodimeric CLOCK:BMAL1 transcriptional activator complex. Science **337**, 189–194 (2012). 22653727 10.1126/science.1222804 PMC3694778

[9] J. Cheng , Accurate proteome-wide missense variant effect prediction with AlphaMissense. Science **381**, eadg7492 (2023). 37733863 10.1126/science.adg7492

[10] K. Jaganathan , Predicting splicing from primary sequence with deep learning. Cell **176**, 535–548.e24 (2019). 30661751 10.1016/j.cell.2018.12.015

[11] Y. Lee , Time-of-day specificity of anticancer drugs may be mediated by circadian regulation of the cell cycle. Sci. Adv. **7**, eabd2645 (2021). 33579708 10.1126/sciadv.abd2645 PMC7880601

[12] S. Cal-Kayitmazbatir, L. J. Francey, Y. Lee, A. C. Liu, J. B. Hogenesch, PSMD11 modulates circadian clock function through PER and CRY nuclear translocation. PLoS ONE **18**, e0283463 (2023). 36961772 10.1371/journal.pone.0283463 PMC10038281

[13] T. Zielinski, J. Hay, A. J. Millar, Period estimation and rhythm detection in timeseries data using BioDare2, the free, online, community resource. Methods Mol. Biol. **2398**, 15–32 (2022). 34674164 10.1007/978-1-0716-1912-4_2

[14] J. E. Rutila , CYCLE is a second bHLH-PAS clock protein essential for circadian rhythmicity and transcription of Drosophila period and timeless. Cell **93**, 805–814 (1998). 9630224 10.1016/s0092-8674(00)81441-5

[15] Y. Xu , Modeling of a human circadian mutation yields insights into clock regulation by PER2. Cell **128**, 59–70 (2007). 17218255 10.1016/j.cell.2006.11.043 PMC1828903

[16] Z. Yang, A. Sehgal, Role of molecular oscillations in generating behavioral rhythms in drosophila. Neuron **29**, 453–467 (2001). 11239435 10.1016/s0896-6273(01)00218-5

[17] R. Allada, S. Kadener, N. Nandakumar, M. Rosbash, A recessive mutant of drosophila clock reveals a role in circadian rhythm amplitude. Embo. J. **22**, 3367–3375 (2003). 12839998 10.1093/emboj/cdg318 PMC165643

[18] G. Biondi, G. McCormick, M. P. Fernandez, The *drosophila* circadian clock gene cycle controls the development of clock neurons. PLoS Genet. **20**, e1011441 (2024). 39432537 10.1371/journal.pgen.1011441 PMC11527286

[19] R. V. Kondratov, R. K. Shamanna, A. A. Kondratova, V. Y. Gorbacheva, M. P. Antoch, Dual role of the CLOCK/BMAL1 circadian complex in transcriptional regulation. FASEB J. **20**, 530–532 (2006). 16507766 10.1096/fj.05-5321fje

[20] G. Li, H. Wang, H. Chen, Association of insulin resistance with polymorphic variants of clock and Bmal1 genes: A case-control study. Clin. Exp. Hypertens. **42**, 371–375 (2020). 31612734 10.1080/10641963.2019.1676769

[21] I. Skrlec, J. Milic, R. Steiner, The impact of the circadian genes CLOCK and ARNTL on myocardial infarction. J. Clin. Med. **9**, 484 (2020). 10.3390/jcm9020484. 32050674 PMC7074039

[22] H. Leu , Association of circadian genes with diurnal blood pressure changes and non-dipper essential hypertension: A genetic association with young-onset hypertension. Hypertens. Res. **38**, 155–162 (2015). 25410879 10.1038/hr.2014.152

[23] Y. Zhu , Testing the circadian gene hypothesis in prostate cancer: A population-based case-control study. Cancer Res **69**, 9315–9322 (2009). 19934327 10.1158/0008-5472.CAN-09-0648 PMC2955869

[24] F. Liu , Association between three polymorphisms in *BMAL1* genes and risk of lung cancer in a northeast Chinese population. DNA Cell Biol. **38**, 1437–1443 (2019). 31580742 10.1089/dna.2019.4853

[25] S. E. Jones , Genome-wide association analyses of chronotype in 697, 828 individuals provides insights into circadian rhythms. Nat. Commun. **10**, 343–7 (2019). 30696823 10.1038/s41467-018-08259-7 PMC6351539

[26] J. Bralten , Genetic underpinnings of sociability in the general population. Neuropsychopharmacology **46**, 1627–1634 (2021). 34054130 10.1038/s41386-021-01044-z PMC8280100

[27] Z. Gu , Association of *ARNTL* and *PER1* genes with Parkinson’s disease: A case-control study of Han Chinese. Sci. Rep. **5**, 15891 (2015). 26507264 10.1038/srep15891 PMC4623766

[28] H. V. Firth , DECIPHER: Database of chromosomal imbalance and phenotype in humans using ensembl resources. Am. J. Hum. Genet. **84**, 524–533 (2009). 19344873 10.1016/j.ajhg.2009.03.010 PMC2667985

[29] M. K. Bunger , Mop3 is an essential component of the master circadian pacemaker in mammals. Cell **103**, 1009–1017 (2000). 11163178 10.1016/s0092-8674(00)00205-1 PMC3779439

[30] R. Singla , Haploinsufficiency of a circadian clock gene Bmal1 (arntl or Mop3) causes brain-wide mTOR hyperactivation and autism-like behavioral phenotypes in mice. Int. J. Mol. Sci. **23**, 6317 (2022). 10.3390/ijms23116317. 35682995 PMC9181331

[31] N. Park , A novel Bmal1 mutant mouse reveals essential roles of the C-terminal domain on circadian rhythms. PLoS ONE **10**, e0138661 (2015). 26394143 10.1371/journal.pone.0138661 PMC4578957

[32] E. L. McDearmon , Dissecting the functions of the mammalian clock protein BMAL1 by tissue-specific rescue in mice. Science **314**, 1304–1308 (2006). 17124323 10.1126/science.1132430 PMC3756687

[33] J. L. Couturier , Parental perception of sleep problems in children of normal intelligence with pervasive developmental disorders: Prevalence, severity, and pattern. J. Am. Acad. Child Adolesc. Psychiatry **44**, 815–822 (2005). 16034284 10.1097/01.chi.0000166377.22651.87

[34] C. Carmassi , Systematic review of sleep disturbances and circadian sleep desynchronization in autism spectrum disorder: Toward an integrative model of a self-reinforcing loop. Front Psychiatry **10**, 366 (2019). 31244687 10.3389/fpsyt.2019.00366 PMC6581070

[35] Y. He , The transcriptional repressor DEC2 regulates sleep length in mammals. Science **325**, 866–870 (2009). 19679812 10.1126/science.1174443 PMC2884988

[36] R. Pellegrino , A novel BHLHE41 variant is associated with short sleep and resistance to sleep deprivation in humans. Sleep **37**, 1327–1336 (2014). 25083013 10.5665/sleep.3924 PMC4096202

[37] L. Xing , Mutant neuropeptide S receptor reduces sleep duration with preserved memory consolidation. Sci. Transl. Med. **11**, aax2014 (2019). 10.1126/scitranslmed.aax2014 PMC7587149 31619542

[38] G. Shi , A rare mutation of beta1-adrenergic receptor affects sleep/wake behaviors. Neuron **103**, 1044–1055.e7 (2019). 31473062 10.1016/j.neuron.2019.07.026 PMC6763376

[39] G. Shi , Mutations in metabotropic glutamate receptor 1 contribute to natural short sleep trait. Curr. Biol. **31**, 13–24 (2020). 33065013 10.1016/j.cub.2020.09.071 PMC12352508

[40] N. Hoang , Sleep phenotype of individuals with autism spectrum disorder bearing mutations in the PER2 circadian rhythm gene. Am. J. Med. Genet. A **185**, 1120–1130 (2021). 33474825 10.1002/ajmg.a.62086

[41] C. Ramanathan, S. K. Khan, N. D. Kathale, H. Xu, A. C. Liu, Monitoring cell-autonomous circadian clock rhythms of gene expression using luciferase bioluminescence reporters. J. Vis. Exp. **67**, 4234 (2012). 10.3791/4234 PMC3490247 23052244

[42] Y. Lee, A. R. Jang, L. J. Francey, A. Sehgal, J. B. Hogenesch, KPNB1 mediates PER/CRY nuclear translocation and circadian clock function. eLife **4**, e08647 (2015). 26319354 10.7554/eLife.08647 PMC4597257

[43] J. Colomb, L. Kaiser, M. Chabaud, T. Preat, Parametric and genetic analysis of drosophila appetitive long-term memory and sugar motivation. Genes Brain Behav. **8**, 407–415 (2009). 19220480 10.1111/j.1601-183X.2009.00482.x

[44] M. J. Krashes, S. Waddell, Rapid consolidation to a radish and protein synthesis-dependent long-term memory after single-session appetitive olfactory conditioning in drosophila. J. Neurosci. **28**, 3103–3113 (2008). 18354013 10.1523/JNEUROSCI.5333-07.2008 PMC2516741

