# Supplementary material for: Rare variants in BMAL1 are associated with a neurodevelopmental syndrome
Source: Proc Natl Acad Sci U S A. 2025 Jul 28;122(31):e2427085122. doi: 10.1073/pnas.2427085122 (PMC12337293; doi:10.1073/pnas.2427085122)
Supplement: Supplementary file 1 — Appendix 01 (PDF) [file pnas.2427085122.sapp.pdf]

PNAS

Supporting Information for
Rare variants in *BMAL1* are associated with a neurodevelopmental syndrome

Vishnu Anand Cuddapah,[1,2]* Dechun Chen,[3,4] Bumsik Cho,[3,4] Rebecca Moore,[3,4] Mohnish Suri,[5] Hana Safraou,[6] Frederic Tran-Mau-Them,[6] Ashley Wilson,[10] Jacqueline Odgis,[11] Atteeq U. Rehman,[10] Carol Saunders,[7] Shiva Ganesan,[8] Vaidehi Jobanputra,[10,12] Stephen W. Scherer,[13] Ingo Helbig,[8,9] Amita Sehgal[3,4]*

[1] Jan and Dan Duncan Neurological Research Institute, Texas Children's Hospital, Houston, Texas, USA
[2] Division of Neurology and Developmental Neuroscience, Department of Pediatrics, Baylor College of Medicine, Houston, Texas, USA
[3] Howard Hughes Medical Institute, University of Pennsylvania; Philadelphia, PA, USA
[4] Chronobiology and Sleep Institute, Perelman School of Medicine, University of Pennsylvania; Philadelphia, PA, USA
[5] Nottingham Clinical Genetics Service, Nottingham University Hospitals NHS Trust, Greater Nottingham, UK
[6] Laboratoire de Génomique médicale, CHU Dijon-Bourgogne, Dijon, France. INSERM UMR 1231, Génétique des Anomalies du Développement, Université de Bourgogne Franche-Comté, Dijon, France
[7] Department of Pathology and Laboratory Medicine, Children's Mercy – Kansas City and Departments of Pediatrics and Pathology, UMKC School of Medicine, Kansas City, MO, USA
[8] Division of Neurology, The Epilepsy NeuroGenetics Initiative (ENGIN), Department of Biomedical and Health Informatics (DBHi), Children's Hospital of Philadelphia, Philadelphia, PA, 19104 USA
[9] Department of Neurology, University of Pennsylvania, Perelman School of Medicine, Philadelphia, PA, 19104 USA
[10] New York Genome Center, New York, NY 10013
[11] Icahn School of Medicine at Mount Sinai, New York, NY 10029
[12] Columbia University Irving Medical Center, New York, NY 10032
[13] The Centre for Applied Genomics, Program in Genetics and Genome Biology, The Hospital for Sick Children, Toronto, ON M5G 0A4, Canada. McLaughlin Centre and Dept. of Molecular Genetics, University of Toronto, Toronto, ON M5S 1A8, Canada.

*Corresponding authors: Vishnu Anand Cuddapah and Amita Sehgal

Email: Vishnu.Cuddapah@bcm.edu and amita@pennmedicine.upenn.edu

This PDF file includes:

Supporting text
Figures S1 to S3
Tables S1 to S5

Supporting Information Text

In individual #1, a 793 kb microduplication involving chromosome 8p11.21-p11.1 was identified and classified as a variant of uncertain significance (Table S4); this includes the *CHRNB3*, *THAP1*, and *RNF170* genes, which are not known to be triplosensitive. Individual #2 exhibited a variant in *CAPN12*, which is not known to be associated with human disease, and a single variant in *KDM5B*, which is known to cause disease (MIM: 618109) in an autosomal recessive pattern(10). Individual #3 exhibited variants in several different genes, including a rare inherited variant in *CREBBP;* however, there were no obvious signs of Menke-Hennekam syndrome (MIM: 618332) or Rubinstein-Taybi syndrome (MIM: 180849), which are associated with pathogenic variants in *CREBBP*. An inherited variant in *SKI* in Individual #3 was not associated with a craniosynostosis syndrome (MIM: 182212) seen with heterozygous pathogenic variants. Likewise, the inherited heterozygous variant in *SLC1A3* in Individual #3 was not associated with episodic ataxia as previously shown with pathogenic variants (MIM: 612656). Pathogenic variants in *COL4A1* are linked to vascular phenotypes not observed in Individual #3. Individual #3 also carries variants in *PTPN23*, *AGMO*, *NAGLU*, and *NARS2*, which are typically associated with autosomal recessive disorders; given that only one variant for each of these genes was identified, these are not thought to contribute to Individual #3's neurodevelopmental syndrome. The maternally inherited *HUWE1* variant of uncertain significance in Individual #5 was considered as a possible diagnosis given significant skewing of X-chromosome inactivation in the mother. However, other hallmark features of the disorder (MIM: 309590) were not present, making the diagnosis challenging. In addition, Individual #5 was found to be a carrier for a variant in *ALDOA*, which is associated with autosomal recessive disease (MIM: 611881). The heterozygous variant in this case was thought to be non-contributory to Individual #5's neurodevelopmental syndrome. A maternally inherited variant was identified in *CACNA1I* in Individual #6 and classified as a variant of uncertain significance; this variant is present in gnomAD 17 times, indicating it is less likely to be the underlying cause of neurodevelopmental delay. Finally, Individual #9 was found to have a heterozygous variant in *NIPA2*, which has not conclusively been implicated in monogenic disorders. In summary, none of the additional variants identified in this cohort are thought to provide an underlying cause for these individuals' clinical syndrome.

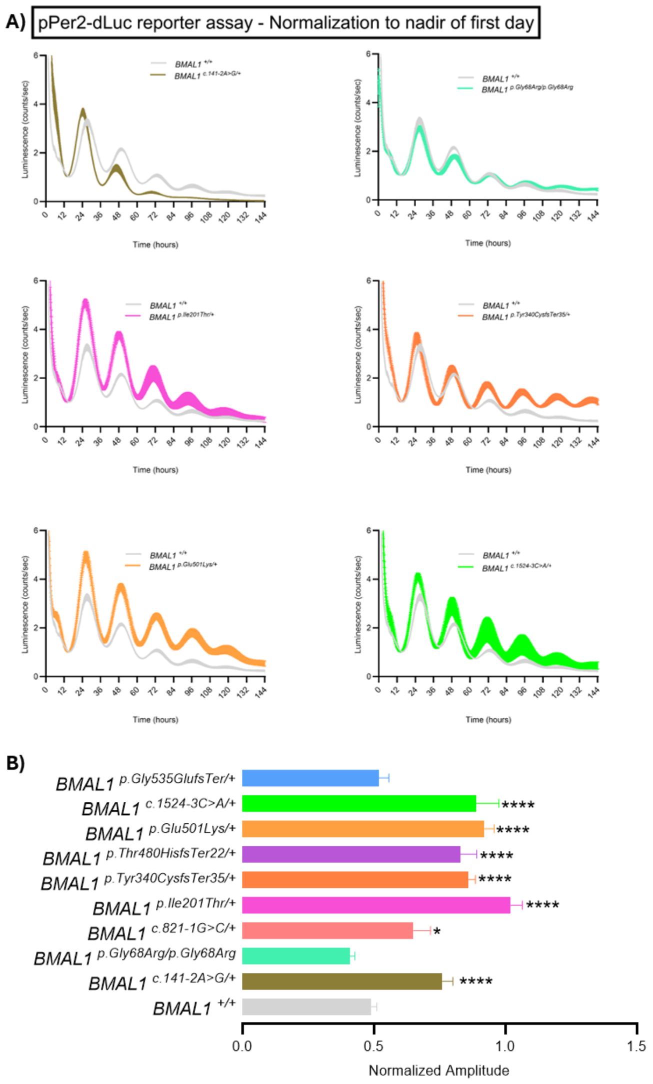

Fig. S1. U2OS cells expressing *Per2*-dLuc reporter and *BMAL1* variants reveal altered BMAL1 function. A) Normalized luminescence after dexamethasone synchronization was recorded for 6 days. Data are normalized to the nadir of the first day. The same genetic control condition *BMAL1*$^{+/+}$ is plotted in each trace to aid in comparison to variants. Traces indicate average values and thickness of the line depicts standard error of the mean. n = 5-9 experiments/condition. **B)** Circadian parameters calculated through BioDare2. One-way ANOVA with Benjamini, Krieger, and Yekutieli's two-stage step-up method to control the false discovery rate with comparisons made to the control genotype. *p<0.05 ****p<0.0001. Data are presented as mean values ± SEM.

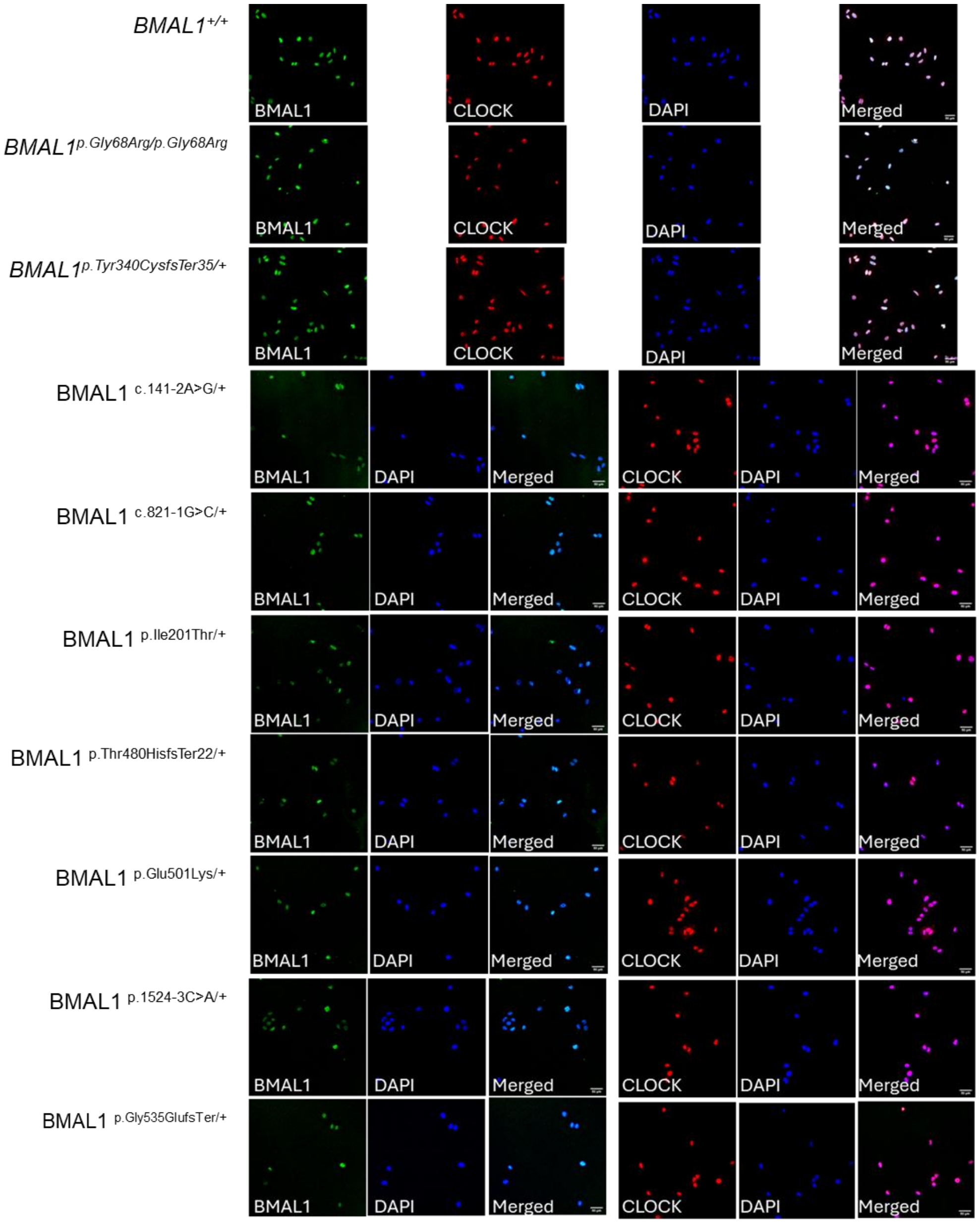

Fig. S2. Cellular localization of BMAL1 and CLOCK in U2OS cells containing wild-type and variant BMAL1. U2OS cells containing wild-type and variant BMAL1 were fixed and subsequently labeled with antibodies targeted against BMAL1 and CLOCK. DAPI was used as a nuclear stain. In all variant lines, BMAL1 and CLOCK appear to be in similar cellular domains as compared to wild-type and continue to co-localize. Scale bar = 50 μm.

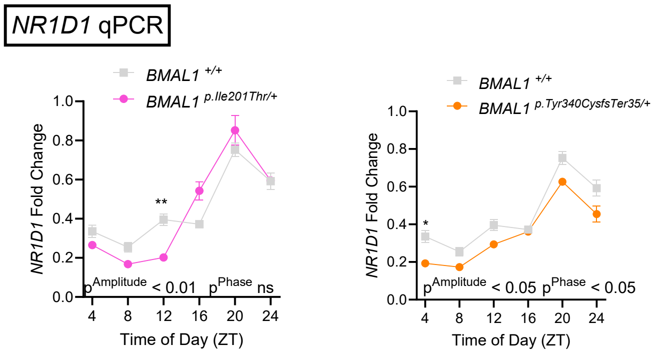

Mixed-effects model: Genetic variant * Timepoint **** Genetics variant x Timepoint ****

Fig. S3. Altered *NR1D1* expression caused by BMAL1 variants. qPCR results *NR1D1* expression at 6 timepoints through the day in wild-type and variant BMAL1 U2OS cells. Daily oscillation of *NR1D1* mRNA in control cell is gray and re-plotted in each trace to allow for comparison to variant lines. Circadian parameters were calculated through BioDare2 and significant results are listed in respective graphs. n = 3 samples/timepoint/condition. One-way ANOVA with Benjamini, Krieger, and Yekutieli's two-stage step-up method to control the false discovery rate for multiple comparisons made to the control genotype. To compare mRNA levels independent of circadian metrics, a mixed-effects model with Geisser-Greenhouse correction and Dunnett's multiple comparisons test were used. *p<0.05 **p<0.01 ***p<0.001. Data are presented as mean values ± SEM.

Table S1. Demographic and genetic features of 10 individuals with *BMAL1* variants reported in this study

	Age at last evaluation	Sex	*BMAL1* Variant	gnomAD frequency (v4.1)	CADD score (v1.3)	Zygosity	Inheritance	Relevant Family History
Individual 1	7 years	male	NM_001297719.2:c.141-2A>G	(1/1,613,980) 6.20e-7	-	Heterozygous	Maternal	Father: ADHD inattentive type<br>Mother: Postpartum depression<br>Older sister: Learning difficulties, infantile spasms/ neonatal seizures<br>Maternal cousin: Motor coordination difficulties and autistic symptoms at 3 years of age<br>Maternal cousin: learning difficulties, social anxiety, depression, and self-harm
Individual 2			NM_001297719.2:c.G202A; p.(Gly68Arg)	0	24.4	Heterozygous	de novo	
Individual 3	12 years	male	NM_001297719.2:c.602T>C; p.(Ile201Thr)	(2/1,614,160) 1.24e-6	23.5	Heterozygous	de novo	
Individual 4	67 years	male	NM_001297719.2:c.821-1G>C	0	-	Heterozygous	Unknown (parents not available for testing)	
Individual 5	13.5 years	male	NM_001297719.2:c.1019_1020del; p.(Tyr340CysfsTer35)	0	-	Heterozygous	de novo	
Individual 6	17 years	male	NM_001297719.2:c.1212dupT; p.(Lys405Ter)	0	-	Heterozygous	Paternal	Father: Learning disability, unable to read and write, tics.<br>Paternal half aunt: Learning disability<br>Paternal grandmother: Depression<br>Maternal cousin: Learning difficulties, ADHD, speech delay
Individual 7	7 years	male	NM_001297719.2:c.1437dupC; p.(Thr480HisfsTer22)	0	-	Heterozygous	de novo	none
Individual 8			NM_001297719.2:c.G1501A; p.(Glu501Lys)	0	23.5	Heterozygous	de novo	
Individual 9	9 years	male	NM_001297719.2:c.1524-3C>A	0	-	Heterozygous	Unknown (sent as singleton)	
Individual 10	2 years	female	NM_001297719.2:c.1604del; p.(Gly535GlufsTer)	0	-	Heterozygous	Unknown (adopted)	

Table S2. Neuropsychiatric features of the 10 individuals with *BMAL1* variants reported in this study

	Developmental delay	Developmental history	Seizure history	Autism spectrum disorder	Sleep or Circadian Disturbance	Sleep History	Neuropsychiatric history
Individual 1	+	Language delay (first single words were at 30 months of age and first phrases at 54 months of age). Average IQ.	-	+	+	Sleep very difficult in early development	-
Individual 2	+	Developmental disorder					
Individual 3	+	GDD, speech apraxia; spoke first words at 3 years of age.	+	+	+	Chronic insomnia	ADHD
Individual 4					-		
Individual 5	+	GDD, severe intellectual disability	+	+	-	No sleep disturbance	Erratic behavioral pattern, hyperactivity with inattention
Individual 6	+	GDD; Motor delay (walked at 21 months of age), Language delay (first single words at 2 years of age. Two-word sentences at 3.5 years of age). Mild delays in fine motor skills with sensory processing difficulties	-	+	+	At 5 years-of-age, only able to sleep a few hours a night continuously; by 17 years of age able to sleep well	ADHD, OCD, Tourette's syndrome
Individual 7				+	-		
Individual 8	+	Developmental disorder					
Individual 9	+	Motor delay, cognitive impairment, poor motor coordination	+	+			
Individual 10	+	GDD	-	(2 years old)	-		-
ADHD: attention-deficit/hyperactivity disorder; FTT: failure-to-thrive; GDD: global developmental delay; NR: not reported; OCD: obsessive-compulsive disorder							

Table S3 continued. Other clinical features of the 10 individuals with *BMAL1* variants reported in this study

	Neuroimaging/ Neurophysiology	Ophthalmological	Cardiovascular	Musculoskeletal	Other features
Individual 1	EEG normal	Not assessed	Not assessed	Toe walking when young	Gastrointestinal: Gastroesophageal reflux disease starting at 2 months of age, frequent constipation
Individual 2					
Individual 3	EEG: Focal slowing left central region				Decreased response to GH stimulation test
Individual 4				Marfan syndrome-like phenotype	
Individual 5	MRI at age 5 years of age showed increased T2 hyperintensity posterior to trigones of both lateral ventricles, likely terminal zones of myelination. Some prominent perivascular spaces.	Right retinal coloboma, high myopia with poor vision both eyes	No heart murmur	Joint hypermobility, hypotonia	BMI 98th centile, penoscrotal hypospadias (repaired) with bilateral undescended testes (treated with bilateral orchidopexy), pubertal delay with low testosterone
Individual 6	Staring spells noted at 3.5 years of age; EEG did not show seizures	Myopia	Innocent murmur noted at 2.5 years of age; electrocardiogram was normal	Tall stature and thin marfanoid habitus; dolichostenomelic features; aching of knees and arms; no evidence of arachnodactyly; not hypermobile based on Beighton score; clinical impression is not suggestive of Marfan syndrome or Ehlers-Danlos syndrome	Asthma; recurrent ear infections requiring myringotomy tubes; non-dysmorphic; cutis marmorata but no striae
Individual 7					
Individual 8					
Individual 9		Exotropia	Heart murmur	Joint laxity, pes planus, hypotonia, ankle weakness	
Individual 10	Not performed	-	-	Duplication of thumb phalanx	FTT, sparse scalp hair (loose anagen)
ADHD: attention-deficit/hyperactivity disorder; FTT: failure-to-thrive; GDD: global developmental delay; GH: growth hormone(s); NR: not reported; OCD: obsessive-compulsive disorder					

Table S4. Additional genetic variants identified for individuals in this study	
Individual 1	8p11.21-p11.1, 793kb DUP including VUS, heterozygous, paternally inherited
Individual 2	*CAPN12:* chromosome 19: 39221631 C>T<br>*KDM5B*: chromosome 1: 202715044 G>C
Individual 3	*CREBBP*, NM_004380.2: c.878T>A; p.(Val293Glu), VUS, heterozygous, inherited<br>*SKI*, NM_003036.4: c.623C>T; p.(Ala208Val), VUS, heterozygous, inherited<br>*PTPN23* (AR), NM_015466.4 c.4258G>C; p.(Glu1420Gln), VUS, heterozygous, inherited<br>*SLC1A3*, NM_004172.5 c.329C>T; p.(Ala110Val), VUS, heterozygous, inherited<br>*AGMO* (AR), NM_001004320.2: c.957_957+18del, LP, heterozygous, inherited<br>*COL4A1*, NM_001845.6: c.1121-986C>T, VUS, heterozygous, de novo<br>*NAGLU* (AR), NM_000263.4: c.2053A>G; p.(Ser685Gly), VUS, heterozygous, de novo<br>*NARS2* (AR), chr11:78476711-78486359 (9.6 Kb deletion involving exons 8-9), LP, heterozygous, inherited
Individual 4	-
Individual 5	*HUWE1:* maternally inherited missense VUS<br>*ALDOA:* chromosome 16: 30078860 G>A
Individual 6	*CACNA1I*: NM_021096.4: c.5664+1G>T; p.?, VUS, heterozygous, maternal
Individual 7	-
Individual 8	-
Individual 9	*NIPA2*: NM_001184889.2: c.1054C>T; p.(Arg352Ter)
Individual 10	-

Table S5. CRISPR/Cas9 reagents used in this study	
BMAL1 Variant	
NM_001297719.2:<br>c.141-2A>G	Guide RNA Sequence UUGUUUUUUCAGAGAAAGCA<br>Guide RNA Cut Location Chr 11: 13,356,744<br>Donor Sequence TTCATGGTACCTTCCATGAGGGTCATCTTTGTCTGTGTCCATACTTTCTCCGAAAAAACAAAAGAGAATGTGACTGCATGTTAATAAAGACATACAGAAGA<br>PCR Primers FOR Primer (5’-3’): AGCTGGTCCAGGTTTTATAGTAAGT<br>PCR Primers REV Primer (5’-3’): CAGACACACAAGGGGACTGG
NM_001297719.1:<br>c.G202A;<br>p.(Gly68Arg)	Guide RNA Sequence AAUAUACAGAACACCAAGGA<br>Guide RNA Cut Location Chr 11: 13,357,077<br>Donor Sequence GCTTTGTAGACAAAATAAGGTCCAAGCTTACCTTGCATTTTTTATCCTTCTTTGATGTTCTGTATATTCTAACCTGGGGGGAGGAAAAAGCAAAAGATAAA<br>PCR Primers FOR Primer (5’-3’): AGCACCCATGTCCTCAACTG<br>PCR Primers REV Primer (5’-3’): GTATGGAGAGCATGGAGGGC
NM_001297719.2:<br>c.602T>C;<br>p.(Ile201Thr)	Guide RNA Sequence UGGCAAUAUCUUUAGGAUGC<br>Guide RNA Cut Location Chr 11: 13,366,703<br>Donor Sequence<br>GAGCCGCTCCCGGGGTGCGGTGTCAGAGGAGGAGAGCTGCTCCTTGACTTTGGCAGTATCTTTAGGGTGCAGGTAGTCAAACAAACTCTGACCAATCAGATCATTCTGGGG<br>PCR Primers FOR Primer (5’-3’): TGGTGTCAGGCAAAGTGGTT<br>PCR Primers REV Primer (5’-3’): GCCCCAGAAAGAGGACAGAG
NM_001297719.2:<br>c.821-1G>C	Guide RNA Sequence CUGCUAGAAAGCAAAGUUCG<br>Guide RNA Cut Location Chr 11: 13,372,149<br>Donor Sequence<br>GGCCAGCTTTTCAAATAGCCTGTGCTGTGGATTGTGCAGAAGCTTTTTCGATCTGGTAGAAAGCAAAGGTCGTGGTTAGTGTCAGCACTAGGTTTGGGCCATGGAGGTGTA<br>PCR Primers FOR Primer (5’-3’): TTTTTGGGGGAGCCTTCCTC<br>PCR Primers REV Primer (5’-3’): AATGCAGTCGTCCAATTGCG
NM_001297719.1:<br>c.1019_1020del;<br>p.(Tyr340CysfsTer35)	Guide RNA Sequence GGAAAUCAGGGUGAAAUCUA<br>Guide RNA Cut Location Chr 11: 13,372,352<br>Donor Sequence TACCTCTGGTCTACAAAAACAAACTTTCCATCTATCGCGTGCCGAGAAACATTCCATGGATTTCACCCTGATTTCCCCGTTCACTGGTTGTGGAACTACA<br>PCR Primers FOR Primer (5’-3’): TACACCTCCATGGCCCAAAC<br>PCR Primers REV Primer (5’-3’): CCACTGCACCCAGCTTTTTC
NM_001297719.2:<br>c.1437dupC;<br>p.(Thr480HisfsTer22)	Guide RNA Sequence AAUCCCUGGAACAGUGGGGU<br>Guide RNA Cut Location Chr 11: 13,378,358<br>Donor Sequence<br>CCTATTTTTCCTGCCCCAGCCCGGGTTCCCCCTGGAATCCCTGGAACAGTGGGGGTGGGTCCTCTTTGGGCCACCTACAACAAAGGAATCTGAGAGTAGGA<br>PCR Primers FOR Primer (5’-3’): TCCCTGCTGGAATGCCTTTT<br>PCR Primers REV Primer (5’-3’): GCGCTGGGCTATTTTGGTTT
NM_001297719.1:<br>c.G1501A;<br>p.(Glu501Lys)	Guide RNA Sequence UGAUUUCCUCAGCAAUCAUU<br>Guide RNA Cut Location Chr 11: 13,378,416<br>Donor Sequence AACAGAGGAACTAGAAGGTGTTACTTGCCTGTGGATTTCCATGATTTCCTTAGCAATCATGCGGCCTATTTTTCCTGCCCCAGCCCGGGTTCCCCCTGGAA<br>PCR Primers FOR Primer (5’-3’): TGGATCATGGGATAAACTGGTT<br>PCR Primers REV Primer (5’-3’): TGGGCTATTTTGGTTTAGCTTCT
NM_001297719.2:<br>c.1524-3C>A	Guide RNA Sequence UCUUUUCUGACAGGAUAAGA<br>Guide RNA Cut Location Chr 11: 13,381,165<br>Donor Sequence ATGTTCAATGGGCTGGAGCCACAGCTAGAAGGCGATGACCCTCTGATCCTTTCAGAAAAGAGAATTACACTGCCTTTTCTTTTTTCAGTGGAGTGTATGTG<br>PCR Primers FOR Primer (5’-3’): GGGCTTTGTCAGTGAGATGC<br>PCR Primers REV Primer (5’-3’): CCCACCCCCAACATTTCAGA

NM_001297719.2:<br>c.1604del;<br>p.(Gly535GlufsTer)	Guide RNA Sequence AGUCUUACCUUCUUGCCUCC<br>Guide RNA Cut Location Chr 11: 13,381,246<br>Donor Sequence CAGCCCATTGAACATCACGAGTACGCCTCCCCCTGATGCCTCTTCTCCAGAGGCAAGAAGGTAAGACTGATGATTCTTAGCCTAAGCTAGAGAACCTCTT<br>PCR Primers FOR Primer (5’-3’): CCCCTTTCTCACCTTTACCCC<br>PCR Primers REV Primer (5’-3’): TCCCTTCTTCCACTCACATGG
